# Debate: Standing up for science – how to combat misinformation in child mental health? Five recommendations for disentangling fact from fiction

**DOI:** 10.1111/camh.70055

**Published:** 2025-12-12

**Authors:** Nina Higson‐Sweeney, Douglas Badenoch, André Tomlin

**Affiliations:** ^1^ Department of Experimental Psychology University of Oxford Oxford UK; ^2^ National Elf Service Minervation Ltd Oxford UK

**Keywords:** Digital literacy, mental health, misinformation, social media, young people

## Abstract

The rising use of digital technologies and social media means that individuals, including young people, are increasingly searching for and consuming health‐related information online. Although there are benefits to this, there has also been an increase in health‐related misinformation, with growing concerns about the impact of this in the context of child mental health. This debate article outlines what misinformation is and why it is an issue in child mental health, before considering the impact that misinformation can have in relation to decision‐making, help‐seeking, medical mistrust, and stigma. Drawing on experiences with science communication, five recommendations are then presented for combating misinformation, focusing on what individuals can do when confronted with misinformation, how to have conversations with others about misinformation, and what can be done to promote digital literacy.

## What is misinformation, and why is it an issue?

Misinformation, referring to incorrect or misleading information, is a widespread issue and is related to the rising use of digital technologies and social media on a global scale. Misinformation is particularly common in the context of physical and mental health, as individuals are increasingly searching for and consuming this type of information online. Health misinformation, defined as health‐related factual claims that do not align with current scientific evidence, is often seen in relation to topics such as vaccines, substance use, infectious diseases, and diet (Suarez‐Lledo & Alvarez‐Galvez, 2021), and was incredibly widespread throughout the COVID‐19 pandemic (Lanier, Diaz, Saleh, Lehmann, & Medford, 2022), heightening the fear and panic already experienced in such an uncertain situation. Mental health mis‐ and disinformation (i.e., deliberate misinformation) is common, too, particularly around topics like suicide, personality disorders, psychosis, and treatment (Hudon et al., 2025). Research has demonstrated that although there is a general awareness about the presence of misleading or false information on social media, adults find it difficult to confidently detect it (Stimpson & Ortega, 2023), highlighting an area of vulnerability when it comes to consuming health information.

Increasingly, there are concerns about misinformation in child mental health. These concerns relate to both the spread of misinformation regarding child mental health, as well as the impact of mental health misinformation on children themselves, and the potential harm and distress it may cause. Like adults, young people are aware of misinformation online and attempt to use different techniques to help verify the reliability of a source (Loades et al., 2025); yet it can also be challenging to determine accuracy, particularly when these sources of information may have other features that indicate credibility. As such, navigating misinformation can feel like a minefield for adults as well as young people, and can have a detrimental impact on the individual as well as broader society.

## What is the impact of misinformation?

Mental health misinformation can have a negative impact in numerous ways. Foremost, it can influence health‐related decision‐making, affecting how individuals perceive and make sense of their experiences, whether help is sought, and what treatments are undertaken (Starvaggi, Dierckman, & Lorenzo‐Luaces, 2024). Based on mental health misinformation, some people may minimise or dismiss their struggles, believing they are not ill enough to be taken seriously, and subsequently not reach out for support. This can lead to symptoms getting worse. Other people may become hypervigilant and seek support when it is not needed, which can be time‐consuming and emotionally distressing. Others may even adopt unsafe health practices, rejecting recommended treatments and opting for untested alternatives, which could exacerbate symptoms or put them at risk.

Furthermore, persistent exposure to mental health misinformation may contribute to distrust in healthcare professionals, with inaccurate information undermining confidence in public healthcare systems (Starvaggi et al., 2024). Distrust can make it harder for people to seek help, but it may also make it more challenging to implement evidence‐based policies.

More broadly, mental health misinformation can lead to an increase in stigma, which refers to a mark or label that provokes prejudice, discrimination, and rejection from others (Hoffner, Salomi, Apkhazishvili, & Edu, 2025). Stigmatising messages are often seen in the media in relation to mental health, suggesting that individuals with mental health conditions are at fault for their problems, are violent or lazy, and will not recover (Hoffner et al., 2025). These messages can be seen as misinformation because they are often inaccurate yet can be internalised by individuals in relation to themselves and others. Not only does stigma work to discourage people from seeking help through fear and shame, but it can also negatively impact relationships, school, work, and leisure, all of which can contribute to poor mental health.

With rates of poor mental health continuing to rise in young people, there is an increased focus on early help‐seeking and timely interventions. But if the young people who need help do not feel able to reach out, do not have trust in the public healthcare systems, and are afraid of the potential stigma they might experience if diagnosed with a mental health condition, it is difficult to understand how this situation can be improved. In light of the potential impact of misinformation around child mental health, there is a clear need to identify strategies to combat it.

## How can professionals combat misinformation?

It is clear why misinformation in child mental health is such a concerning issue, but what is less clear is how this is addressed. As science communicators who work for The Mental Elf and are experienced in disseminating evidence‐based information without bias or spin, we want to share five recommendations for combating misinformation. The goal of these recommendations is to help guide professionals when faced with misinformation and to have conversations about misinformation with young people, parents, teachers, and other stakeholders in child mental health.

### Recommendation 1: Be curious and inquisitive

When faced with new information, it is important for individuals to become their own investigators and work to identify whether the information can be considered reliable. This could involve reading beyond the headline to the content itself, cross‐checking the information across different (reputable) sources, and conducting literature reviews to determine whether there is robust scientific evidence to support the claims made. Questions to keep in mind include: Who has shared this information, and why are they sharing it? Do they have relevant qualifications or expertise? Is this information current or outdated? Is it shared or supported by a credible organisation (e.g., World Health Organization)? What platform has it been shared on, and why was this platform chosen? Is the language neutral and balanced, or is it emotional and sensational? Through being inquisitive and not taking information at face value, there is a lot to be gleaned about the reliability of a piece of information, which may help to determine whether or not it is misinformation.

### Recommendation 2: Have open and honest conversations

If approached by someone who is sharing misinformation, believes a piece of misinformation, or is concerned about information they have heard or read, one of the best things professionals can do is have an open and honest conversation. This advice holds for young people, too. Providing a safe environment for the conversation and making use of existing skills such as active listening may help to understand the individual's perspective, demonstrate that they are being taken seriously, and allow for an opening to share alternative perspectives or different information. As part of this, it is important to be aware of the potential power dynamics within the conversation. It might not be possible to change this, but awareness may help the navigation of the conversation and aid psychological safety for all involved parties.

### Recommendation 3: Work together

Related to the above, there may be opportunities for collaborative working and problem‐solving to identify how reliable or credible a piece of information is. This approach could be quite empowering, particularly if working with young people, who really value agency and autonomy.

### Recommendation 4: Use available tools

When in doubt, there are freely available, trustworthy tools that can help determine whether information is factually sound. For example, the charity Sense About Science launched the Ask for Evidence campaign in 2011. This national campaign encouraged thousands of individuals to actively question and ask for evidence when faced with information that did not appear to be reliable. Their website includes guides, templates, and videos that help individuals ask others for evidence, or to assess the evidence themselves (Sense About Science, 2025). Promoting resources like this and signposting others to these resources is an important part of helping to combat misinformation.

### Recommendation 5: Promote digital literacy

While there is a need for policy‐level change regarding how mental health misinformation is tackled, there are small steps that any individual can take to help promote digital literacy. This includes: helping others to identify misinformation; sharing reliable, trusted information; pausing before reposting; modelling critical thinking; becoming a trusted source; sharing resources and tools with others; continuing to engage in education and upskilling in relation to misinformation; and engaging with science communication.

## Conclusion

Misinformation regarding child mental health is a growing concern, and much work is needed to understand how it can be adequately addressed. However, there are steps that professionals can take to help themselves and others disentangle fact from fiction, including open and honest conversations, collaborative problem‐solving, and continued upskilling in digital literacy.

## Funding information

The Mental Elf (part of National Elf Service) is funded by a Wellcome Trust Discretionary Award.

## Declaration of interests

The authors work for The Mental Elf (part of National Elf Service), which is a website that focuses on disseminating the latest evidence‐based research, policy, and guidance.

## Ethical information

No ethics approval was required for this debate article.

## Data Availability

Data sharing not applicable to this article as no datasets were generated or analysed during the current study.
